# Nepali Older Adults With Pre-existing Conditions and Their Healthcare Access Amid COVID-19 Pandemic

**DOI:** 10.1093/geroni/igab046.3417

**Published:** 2021-12-17

**Authors:** Aman Shrestha, Saruna Ghimire, Bunsi Chapadia, Uday Narayan Yadav, Sabuj Kanti Mistry, Om Prakash Yadav, Mark Fort Harris

**Affiliations:** 1 Miami University, Oxford, Ohio, United States; 2 University of New South Wales, SYDNEY, New South Wales, Australia; 3 University of New South Wales, Kensington, New South Wales, Australia; 4 Ministry of Health and Population, Kathmandu, Bagmati, Nepal

## Abstract

COVID-19 has greatly impacted older adults with pre-existing non-communicable conditions (hereafter called pre-existing conditions) in terms of their access to essential healthcare services. Based on the theory of vertical health equity, this study investigated access to healthcare by Nepali older adults with pre-existing conditions during the COVID-19 pandemic. A cross-sectional study surveyed 847 randomly selected older adults (≥60 years) in three districts of eastern Nepal. Survey questionnaire, administered by trained community health workers, collected information on participants reported difficulty obtaining routine care and medications during the pandemic, in addition to questions on demographics, socioeconomic factors, and pre-existing conditions. Cumulative scores for pre-existing conditions were recoded as no pre-existing condition, single condition, and multimorbidity for the analyses. Chi-square tests and binary logistic regressions determined inferences. Nearly two-thirds of the participants had a pre-existing condition (43.8% single condition and 22.8% multimorbid) and reported experiencing difficulty obtaining routine care (52.8%) and medications (13.5%). Participants with single (OR: 3.06, 95%CI: 2.17-4.32) and multimorbid (OR: 5.62, 95%CI: 3.63-8.71) conditions had three and five-fold increased odds of experiencing difficulty accessing routine care. Findings were similar for difficulty obtaining medication (OR single: 3.12, 95%CI: 1.71-5.69; OR multimorbid: 3.98, 95%CI: 2.01-7.87) where odds were greater than three-folds. Older adults with pre-existing conditions in Nepal, who require routine medical care and medication, faced significant difficulties obtaining them during the pandemic, which may lead to deterioration in their pre-existing conditions. Public health emergency preparedness should incorporate plans for both managing the emergency and providing continuing care.

